# The power spectrum and functional connectivity characteristics of resting-state EEG in patients with generalized anxiety disorder

**DOI:** 10.1038/s41598-025-90362-z

**Published:** 2025-02-18

**Authors:** Hangwei Wang, Shaoqi Mou, Xuedan Pei, Xiaomei Zhang, Shanhong Shen, Jianfeng Zhang, Xinhua Shen, Zhongxia Shen

**Affiliations:** 1 Key Laboratory of Psychiatry, Huzhou Third Municipal Hospital, The Affiliated Hospital of Huzhou University, Huzhou, 313000 People’s Republic of China; 2 Sleep Medical Center, Huzhou Third Municipal Hospital, The Affiliated Hospital of Huzhou University, Huzhou, 313000 People’s Republic of China; 3 Qingdao Mental Health Center, Qingdao, 266034 People’s Republic of China; 4 Jifu Hospital, Xuzhou, 221112 People’s Republic of China

**Keywords:** Generalized anxiety disorder (GAD), Electroencephalogram (EEG), Power spectrum, Functional connectivity (FC), Alpha asymmetry, Anxiety, Biomarkers

## Abstract

Recent studies have suggested a relationship between abnormal neurophysiological functions and generalized anxiety disorder (GAD). However, studies on its electrophysiological characteristics, such as its power spectrum and functional connectivity are relatively few and scattered than those on other mental disorders (e.g., depression, ADHD, etc.). The present study aims to reveal the multidimensional electrophysiological characteristics of GAD via comparative analysis of electroencephalogram (EEG) data between GAD patients and healthy controls. Specifically, resting-state EEG, with a duration of 10 min, was recorded from 98 GAD patients and 92 healthy control participants. The electrophysiological characteristics, including the power spectrum, alpha asymmetry, and functional connectivity, were extracted and compared between the two groups. The results revealed significantly increased beta-band activity; decreased ipsilateral fronto-temporal and parieto-temporal functional connectivities in the lower frequency bands (theta–beta band); as well as decreased frontal‒parietal and frontal‒occipital connectivities in the higher frequency bands (beta‒gamma band) in GAD patients. Additionally, alpha asymmetry analysis revealed a significantly greater rightward temporal alpha asymmetry in GAD patients. These findings suggest the existence of significant EEG characteristics in patients with GAD, supporting previous conclusions regarding abnormal neurophysiological functions in psychiatric disorders and potentially leading to the identification of biomarkers for clinical diagnosis.

**Supplementary Information:**

The online version contains supplementary material available at 10.1038/s41598-025-90362-z.

## Introduction

Generalized anxiety disorder (GAD) is a subtype of anxiety disorder characterized by frequent and excessive anxiety and uncontrollable worry about a variety of topics^1,2^. These symptoms often lead to overmuch worry about real-life problems and increased painful feelings for patients^3^, impairing their quality of daily life and associated with improved risk for future issues^4^. With the development of modern society, the incidence rate of GAD is increasing. According to previous studies, the prevalence of GAD is 3.1% per year or 5.7% over a lifetime in the United States of America^5^, whereas the prevalence is 5.3% in urban China^6^ and 6% in the United Kingdom^7^. While many advancements have been made in understanding the physiological and psychological mechanisms of GAD through previous studies, research on the resting-state EEG characteristics of GAD is relatively limited compared with that of other psychiatric disorders (e.g., schizophrenia, depression, ADHD, etc.)^8^. To provide more supportive evidence for establishing unified EEG features of GAD and determining biological markers to assist in clinical diagnosis, the present study aims to reveal the EEG characteristics of GAD via three analytic methods: power spectrum, alpha asymmetry, and functional connectivity analyses.

EEG is an important electrophysiological measurement technique that records brain electrical activity through electrodes placed on the participant’s scalp. Owing to its noninvasiveness, low cost, ease of use, and high temporal resolution, EEG has been widely used to reveal the neurophysiological features of various mental disorders^8^. Power spectral analysis is one of the most commonly used methods for analyzing EEG data, which can reveal the energy distributions of various frequency components in brain electrophysiological activity^9^. In studies of anxiety disorders, the EEG power spectrum analysis has revealed distinct abnormal activities in patients compared with healthy controls. For example, Shadli et al.^10^ reported that patients with anxiety disorders presented significantly increased theta rhythms in the right frontal area during anxiety evoked by a stop-signal task. Other resting-state studies have suggested that the increased power of the beta rhythm is related to a high level of anxiety in healthy participants^11,12^ and patients with social anxiety^13^. In recent years, some studies have reported resting-state EEG power spectrum features of GAD, including increased beta-band activity and decreased alpha-band activity^14^, as well as increased beta and gamma-band activity in frontal channels^15^. Combining the above findings, we assumed that the electrophysiological activity of GAD patients should be greater than that of healthy controls, especially in the beta band. As most of the studies obtained those findings via machine learning methods based on relatively small sample sets^14–16^, the present study aims to support and explore the findings in this area with a relatively larger dataset.

On the basis of the EEG power spectrum, alpha asymmetry is commonly used to investigate brain lateralization. Alpha asymmetry refers to different hemispheric distributions of electrophysiological activity across cortical areas of the brain and it is conceptualized as the relative hemispheric difference in alpha power (8–13 Hz)^17^. Resting-state alpha power asymmetry in frontal or parietal areas has been reported in various studies of anxiety, such as higher right-than-left fronto-lateral alpha power in patients with panic disorder^18^, social anxiety disorder^19^, or general anxiety disorder^20^. Notably, alpha power is inversely related to cortical activity^21^; thus, higher right-than-left alpha power indicates greater left-than-right activity. According to the framework of the valence‒arousal model^22^, anxious apprehension (i.e., worry, which is a characteristic of obsessive-compulsiveness, generalized anxiety states, and trait anxiety) and anxious arousal (physiological hyperarousal and somatic tension, often caused by panic attacks and high-stress situations) are associated with different patterns of regional brain activity, with more left frontal brain activity indicating anxious apprehension and right parieto-temporal brain activity indicating anxious arousal. Expanding on previous findings of the frontal alpha asymmetry, the present study analyzed the GAD alpha asymmetry features in various brain regions, including the frontal, parietal, occipital, and temporal regions. In addition to the frontal area, we also expected to discover rightward alpha asymmetry in other brain regions, which would reveal the asymmetric brain activity of GAD on a larger spatial scale.

Functional connectivity analysis is another EEG analytical method that has received much attention in recent years. It aims to reveal the patterns of interaction within and between different brain regions via their EEG signals. Compared with other widely used imaging techniques such as functional magnetic resonance imaging (fMRI), EEG recording has high temporal resolution and is thus suitable for describing both spatial and temporal features of neural activation and connectivity^23^. Previous fMRI studies have revealed several functional connectivity characteristics of GAD patients, such as stronger functional connectivity in the amygdala, insula, putamen, thalamus, and posterior cingulate cortex but weaker connectivity in the frontal and temporal lobes^24^; as well as enhanced resting-state functional connectivity in the inferior frontal gyrus, and reduced functional connectivity in the superior temporal gyrus^25^. However, compared with functional connectivity studies on other psychiatric disorders (e.g., depression^26^, social anxiety disorder^27^, etc.), EEG studies of functional connectivities in GAD patients are relatively rare. A previous study using machine-learning analysis suggested that GAD patients have decreased functional connectivity between frontal lobes and other brain regions^14^. The present study aims to support and expand the understanding of the resting-state EEG functional connectivity features of GAD patients. We assumed that abnormal functional connectivity between various brain regions should be a significant characteristic of GAD.

Overall, EEG is an important electrophysiological measurement technology that contains rich information (e.g. power, frequency, and phase) about mental states, and has various potential characteristics for analysis. In the studies of GAD, some characteristics have been discussed, but no unified conclusion has been reached (e.g. power spectrum), and some other characteristics have not been extensively studied (e.g. functional connectivity). Aiming to provide more supporting evidence for establishing a unified EEG characteristics of GAD, this study analyzed the multidimensional resting-state EEG characteristics of patients with generalized anxiety disorder. It examines several aspects, including the power spectrum, alpha asymmetry, and functional connectivity. The power spectrum and alpha asymmetry features were extracted via fast Fourier transform and comparative analysis between the GAD and HC groups to determine the electrophysiological features of GAD patients. Functional connectivities were estimated with two measurements: the coherence coefficient and pairwise phase consistency (PPC). The coherence coefficient measures the strength of the network interaction between electrodes or regions by estimating both the amplitude and phase consistency at each frequency bin via the fast Fourier transform^28^. The pairwise phase consistency (PPC) is an improved measurement of the phase-locking value (PLV). It estimates rhythmic synchronization between two neuron signals from separate sources, and its sample estimator is consistent and not biased by the sample size^29^. On the basis of the previous findings of brain overactivation in anxiety patients, we assume that GAD patients should have increased electrophysiological activity compared with healthy controls, especially in the beta band. Alpha asymmetry analysis should reveal rightward alpha asymmetry, which means greater left-than-right hemisphere activity in GAD patients, especially in the frontal or parietal lobes. Additionally, functional connectivity analysis should reveal the abnormal connectivities between brain regions in GAD patients.

## Methods

### Participants

Two groups of participants were recruited for this study: patients diagnosed with generalized anxiety disorder (GAD) and healthy control participants (HCs). 98 patients (29 males and 69 females, with a mean age of 43.4 ± 10.8 years) who met the Diagnostic and Statistical Manual of Mental Disorders 5 (DSM-5) criteria for GAD were recruited from Huzhou Third People’s Hospital. These patients completed two questionnaires, the 14-item Hamilton Rating Scale for Anxiety (HAMA-14) and the 17-item Hamilton Rating Scale for Depression (HAMD-17), and were required to meet specific criteria: HAMA-14 scores ≥ 14 and HAMD-17 scores < 17. Another 92 healthy participants (37 males and 55 females, with a mean age of 43.5 ± 12.2 years) were recruited from the local community and were assessed by a psychiatrist via the Structural Clinical Interview for the DSM-5. These participants also completed the HAMA-14 and HAMD-17 questionnaires and met specific criteria: HAMA-14 scores < 7 and HAMD-17 scores < 7. Furthermore, all the participants were required to be right-handed, have no other mental disorders (except GAD) or physical disorders, have no history of alcohol or substance abuse, and have no signs of brain damage, as determined by self-reports and medical histories. Additionally, participants were required not to stay up late, consume alcohol or drugs within one day before the test, and not to smoke or consume coffee or tea within 8 h before the EEG recording.

The experiment was approved by the Ethics Committee of Huzhou Third Municipal Hospital. All the experiments were performed in accordance with the relevant guidelines and regulations, and all the participants provided written informed consent before the test. The sample size was calculated via G*power with a two-tailed independent t-test. Based on a medium effect size (d = 0.5), α = 0.05, and 90% power, the minimum sample size was determined at 86 participants for each group. The demographic and clinical characteristics of the participants are presented in Table 1.

**Table 1:** The demographic and clinical characteristics of the participants.

Characteristics	GAD (n = 98)	HC (n = 92)	t	χ^2^	*p* Value
Age (years)	43.4 ± 10.8	43.5 ± 12.2	− 0.049	–	0.961
Gender: male/female	29/69	37/55	–	3.408	0.065
Education (years)	10.6 ± 4.1	10.5 ± 4.9	0.189	–	0.850
HAMA-14 score	19.9 ± 3.8	1.6 ± 1.2	44.408	–	< 0.001
HAMD-17 score	12.7 ± 2.8	3.1 ± 1.8	28.045	–	< 0.001

Age, education, HAMA-14 score, and HAMD-17 score factors were compared between the GAD and HC groups via t-test. The gender ratio was compared between groups with a Chi-square test. All the analyses were conducted at the 0.05 level of significance.

### EEG acquisition and preprocessing

**Fig. 1:**
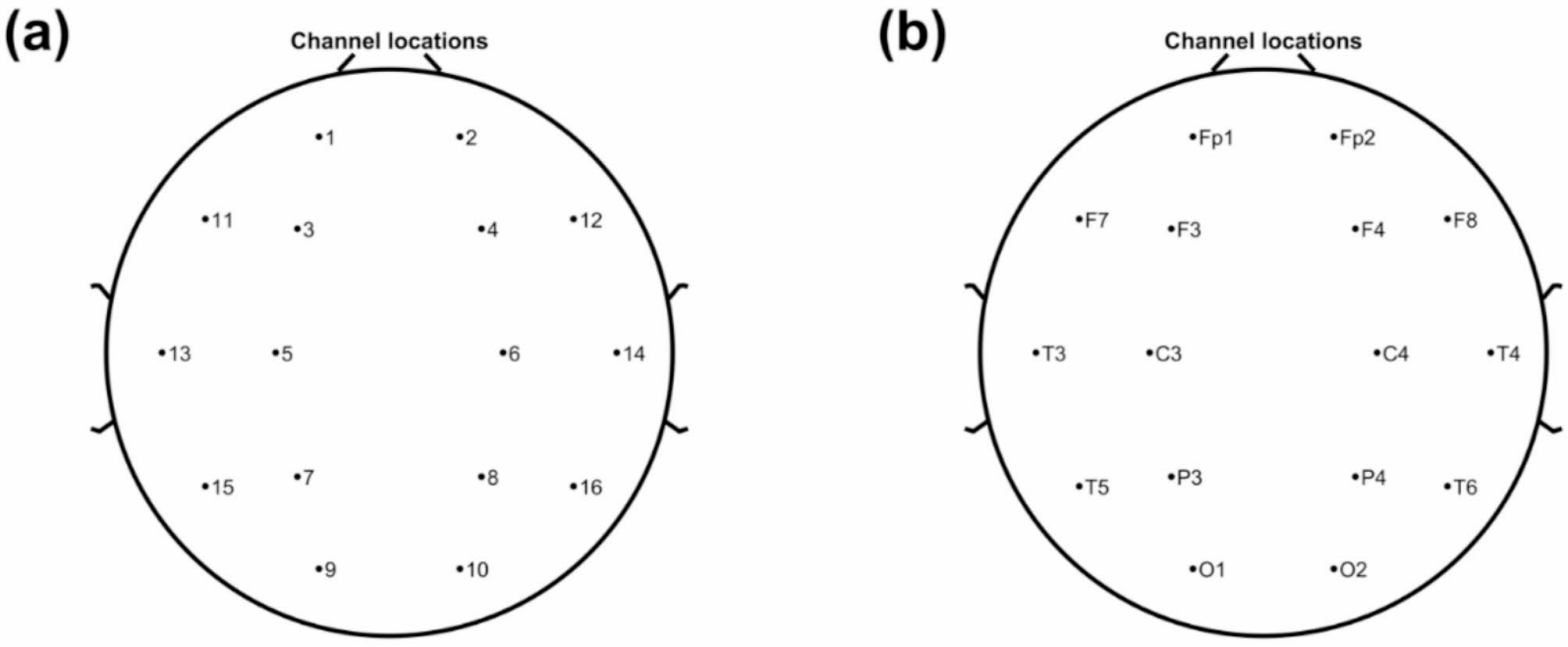
Channel locations of the EEG recording. The channel locations were plotted by (**a**) channel numbers or (**b**) channel names according to the international 10–20 system.

The 16-channel EEG (Fp1, Fp2, F3, F4, C3, C4, P3, P4, O1, O2, F7, F8, T3, T4, T5, and T6, according to the international 10–20 system, Fig. 1) of the participants were recorded by an EEG apparatus (Nicolet EEG TS215605), with a sampling rate of 250 Hz. The impedances of all electrodes were kept below 5 kΩ. Each participant was required to close their eyes, be awake, and relax for ten minutes to record their resting-state EEG. The experiment was conducted in a professional EEG lab at Huzhou Third Municipal Hospital.

The EEG was preprocessed using MATLAB scripts and the EEGLAB toolbox before further analysis. Initially, the raw data were bandpass filtered from 1 to 60 Hz via a fourth-order Butterworth filter, and notch filtered from 49 to 51 Hz to eliminate line-power interference^30^. The data were subsequently rereferenced to the average of the left and right mastoids (A1&A2). Channels with high noise were interpolated by nearby channels, and periods with high noise were rejected via the EEGLAB toolbox. Events with a 4-s gap between each other were imported, and epochs with a length of 4 s were extracted for the stability of functional connectivity measurements^31^. After the epoching, independent component analysis (ICA) was applied to extract 16 principal components, and artifacts (eye blinks, eye movements, head movements, etc.) were rejected.

### EEG analyses and statistics

The analysis of EEG focused on three features: the power spectrum, alpha asymmetry index, and functional connectivity (FC). EEG analysis and statistics were performed via MATLAB scripts in conjunction with the FieldTrip toolbox^32^.

#### Power spectrum analysis

The frequency-domain power spectra of the channels in each trial were calculated via fast Fourier transform (FFT) and averaged across trials, resulting in a multichannel power spectrum (channels × frequency) for each participant. The frequency resolution of the FFT was set at 1 Hz, the temporal resolution was set at 100 ms, and the data were tapered via a 1000 ms Hanning window before the FFT^33–35^. For statistical analyses, an independent samples t-test between the GAD and HC groups was conducted. The result was corrected for multiple comparisons via a cluster-based permutation test, with the significance level of correction set at α = 0.05 (two-tailed), the distance for defining electrodes as neighbors set at 50 mm, and the number of permutations set at 5000^36^. The cluster-based permutation test is a nonparametric statistical method used to detect significance and clustered effects in EEG and is less conservative than the false discovery rate (FDR) or Bonferroni correction, especially when the sample size is large^37^.

#### Alpha asymmetry analysis

For the alpha asymmetry analysis, the averaged alpha-band EEG power was extracted from the channels of each participant with the same FFT as above, except the frequency band was set at 8–13 Hz. Because the frequency resolution was set at 1 Hz, the result is equivalent to the alpha band power density. Four pairs of channels in the contralateral brain regions were selected to estimate the right-versus-left alpha asymmetries, that is: F4–F3 for the frontal area, P4–P3 for the parietal area, O2-O1 for the occipital area, and (T4 + T6) − (T3 + T5), which refer to the difference between the average alpha power of channels T4 and T6 versus to the average alpha power of channels T3 and T5 for the temporal area. Repeated-measures one-way ANOVA of alpha-band power densities of these electrode pairs was performed across participants for each group, and post-hoc analyses with multiple comparisons via the two-stage linear step-up procedure^38^ were conducted between the electrode pairs to estimate their alpha asymmetries within groups. The hemisphere and location of the electrodes were defined as within-subject factors. For the comparison between the groups, the alpha asymmetry was estimated with the laterality coefficient (LC), which indices relative right versus left-sided alpha power via the following formula:

1 $$LC = \frac{{(P_{R} - P_{L} )}}{{(P_{R} + P_{L} )}},$$

where the $P_{R}$ is the average alpha power of the electrodes on the right hemisphere and $P_{L}$ is the average alpha power of the electrodes on the left hemisphere. The LC estimate of alpha asymmetry is highly correlated with the often-used logarithm difference estimation:

2 $$AAI = \ln (P_{R} ) - n(P_{L} ),$$

and the LC has a more intuitive interpretation as an untransformed variable^21,39^. The LCs of brain regions, including the frontal, parietal, occipital, and temporal regions, were calculated for each participant and then compared between groups with the Kruskal‒Wallis rank-sum test. All the analyses were conducted at the 0.05 level of significance, and multiple comparisons were corrected by FDR correction.

#### Functional connectivity analysis

The functional connectivity across channels was estimated via two algorithms: the coherence coefficient and pairwise phase consistency (PPC). The coherence coefficient estimates the linear relationship between two wave functions as a function of frequency by calculating their cross-power spectral density:

3 $$COH_{{{\text{xy}}}} \left( f \right) = \frac{{\left| {S_{xy} \left( f \right)} \right|^{2} }}{{S_{xx} \left( f \right)S_{yy} \left( f \right)}},$$

where $S_{{{\text{xx}}}} (f)$ and $S_{{{\text{yy}}}} (f)$ are the power spectral densities of wave functions ${\text{x}}(t)$, ${\text{y}}(t)$, and where $S_{{{\text{xy}}}} (f)$ denotes the cross-power spectral density between them.

Pairwise phase consistency measures the consistency of phase relationships between neuronal signals within a specific frequency band by computing the phase differences between each pair of signal samples:

4 $$\gamma = \frac{2}{N(N - 1)}\sum\limits_{j = 1}^{N - 1} {\sum\limits_{k = (j + 1)}^{N} {f(\theta_{j} ,\theta_{k} )} },$$

where the function $f$ represents the phase differences by computing the dot product between two unit vectors, expressed as:

5 $$f(\phi ,\omega ) = cos(\phi )cos(\omega ) + sin(\phi )sin(\omega ).$$

This method addresses the issue of overestimating population statistics for sample sizes in phase locking value or spectral coherence methods that may introduce bias when comparing conditions that differ in the number of trials^29^.

The coherence coefficient and PPC values between channels were calculated for each participant in each 1-Hz-length frequency bin and then compared between the GAD and HC groups via an independent samples t-test. The results underwent multiple comparisons corrected with the FDR correction, with the significance level set at α = 0.05 (two-tailed). The results were divided into four frequency bands, including theta (4–7 Hz), alpha (8–13 Hz), beta (14–30 Hz), and gamma (31–60 Hz) bands. The numbers of frequency bins with significant differences between groups were calculated for the functional connectivity between each pair of channels and normalized in each frequency band with the following formula:

6 $$N_{normalized} = \frac{N}{{N_{\max } }},$$

where $N$ is the number of frequency bins with significant differences between groups of functional connectivity between a specific pair of channels, and $N_{{{\text{max}}}}$ is the largest number of frequency bins with significant differences between groups of functional connectivities between all the pairs of channels in the frequency band. The results were presented via the BrainNet toolbox^40^.

## Results

### Power spectrum analysis

**Fig. 2:**
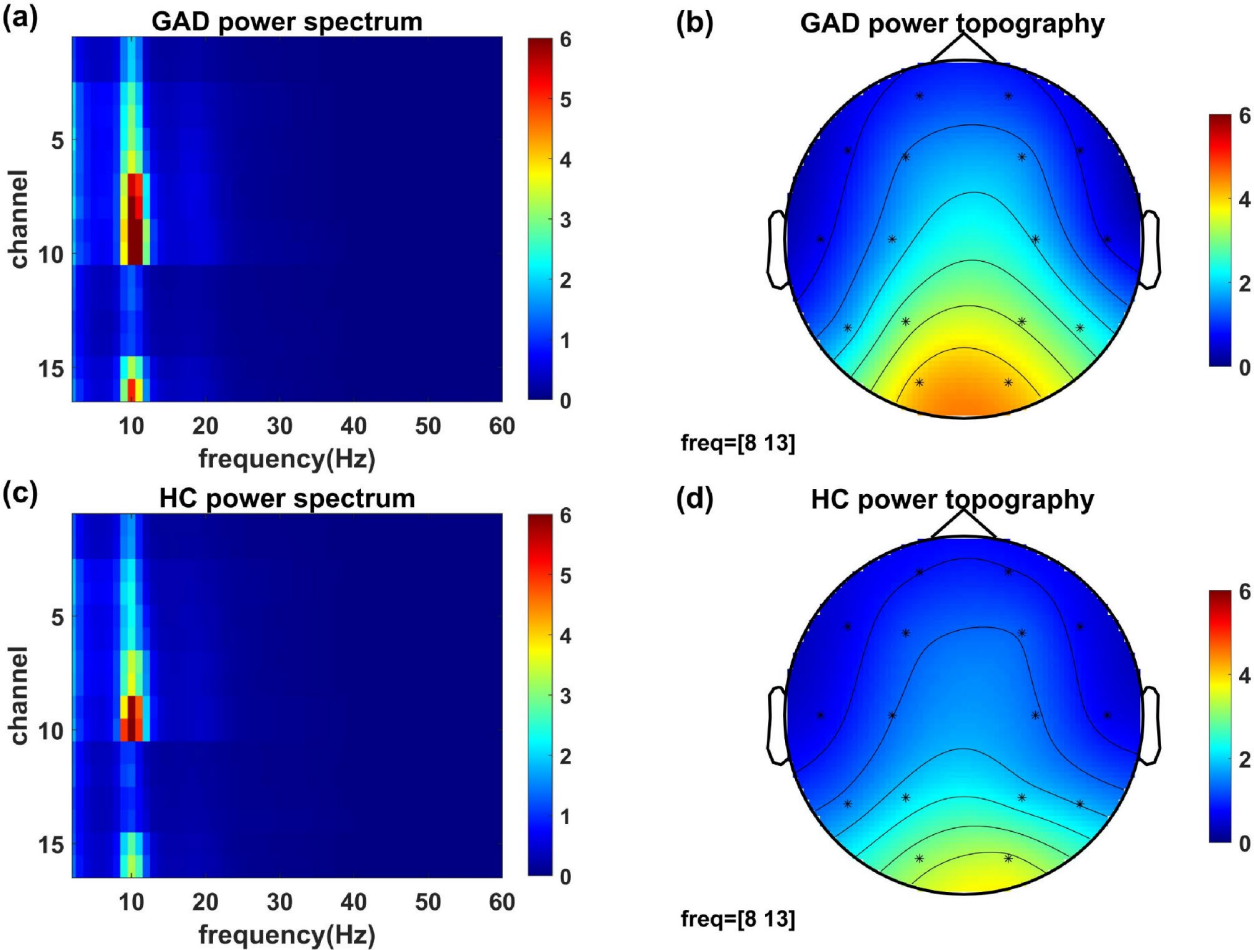
EEG power spectra and topographies of the GAD and healthy control groups. (**a**) Power spectrum of the GAD group. Red indicates the higher power of electrophysiological activity. (**b**) Topography represents the average spatial distribution of the alpha band (8–13Hz) EEG power of the GAD group. Red indicates the higher power of electrophysiological activity. (**c**) Power spectrum of the healthy control group. Red indicates the higher power of electrophysiological activity. (**d**) Topography represents the averaged spatial distributions of the alpha band (8–13 Hz) EEG power of the healthy control group. Red indicates the higher power of electrophysiological activity.

As the first step of power spectrum analysis, the spatial‒frequency domain EEG power spectra of the GAD and healthy control groups were calculated (Fig. 2). The power spectra and topographies revealed posterior dominant rhythms (8–13 Hz) in both groups, which is a significant characteristic of resting-state EEG.

**Fig. 3:**
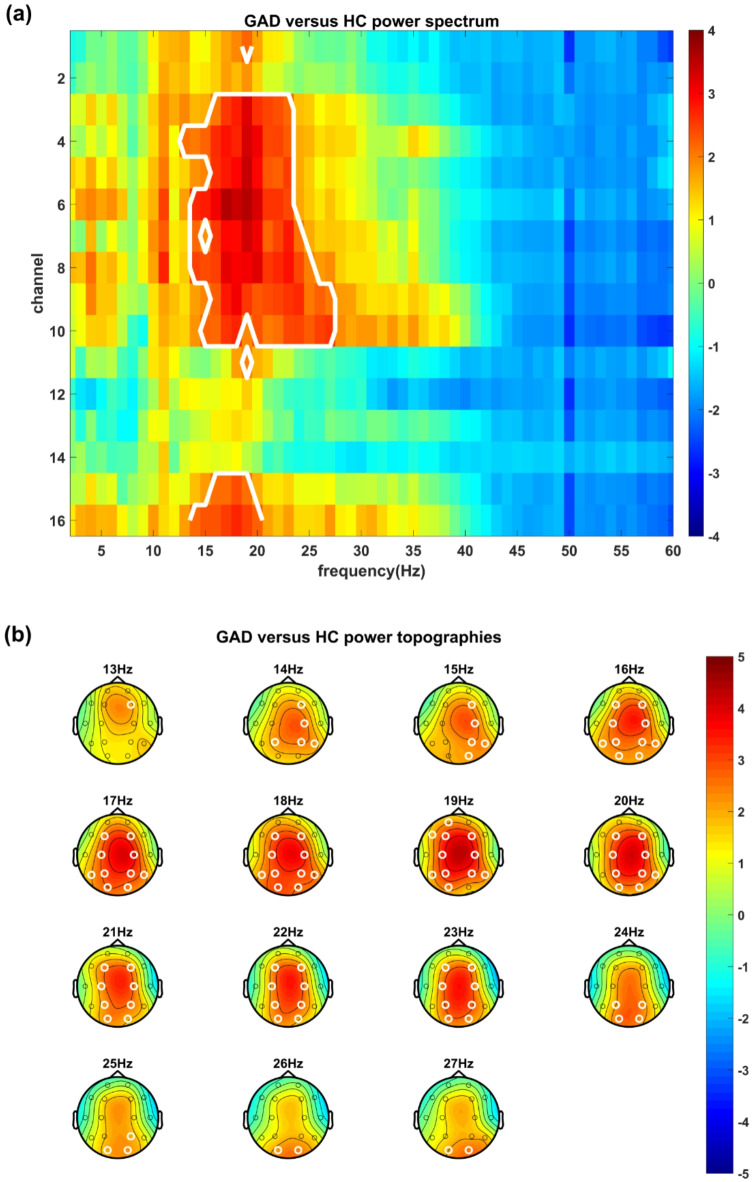
Power spectrum analysis results comparing the GAD and healthy control groups. (**a**) T-statistics of comparing power spectra between the GAD and healthy control groups. The X-axis represents the frequency bins, and the Y-axis represents the channels (for the locations of the channels, please check Fig. 1). The color bar denotes the t value, with red indicating higher power and blue indicating lower power in the GAD group than in the healthy control group. The white lines mark the clusters with significant differences between the groups after cluster-based permutation testing (*p* < 0.05). (**b**) Topographic distribution of the t-statistics. The color bar denotes the t value, with yellow indicating higher power and blue indicating lower power in the GAD group than in the healthy control group. The white circles highlight the channels in the cluster with significant differences between the groups (*p* < 0.05).

A t-test comparing the power spectra of the two groups revealed significant differences in electrophysiological activity between GAD patients and healthy participants. In the beta band (13–27 Hz), GAD patients had significantly higher EEG power than healthy controls (*p* = 0.012, after cluster-based permutation test), indicating excessive brain activity among GAD patients (Fig. 3a). This heightened activity was widely distributed throughout the central axis in the low-beta band and shifted toward the posterior region as the frequency increased (Fig. 3b).

**Fig. 4:**
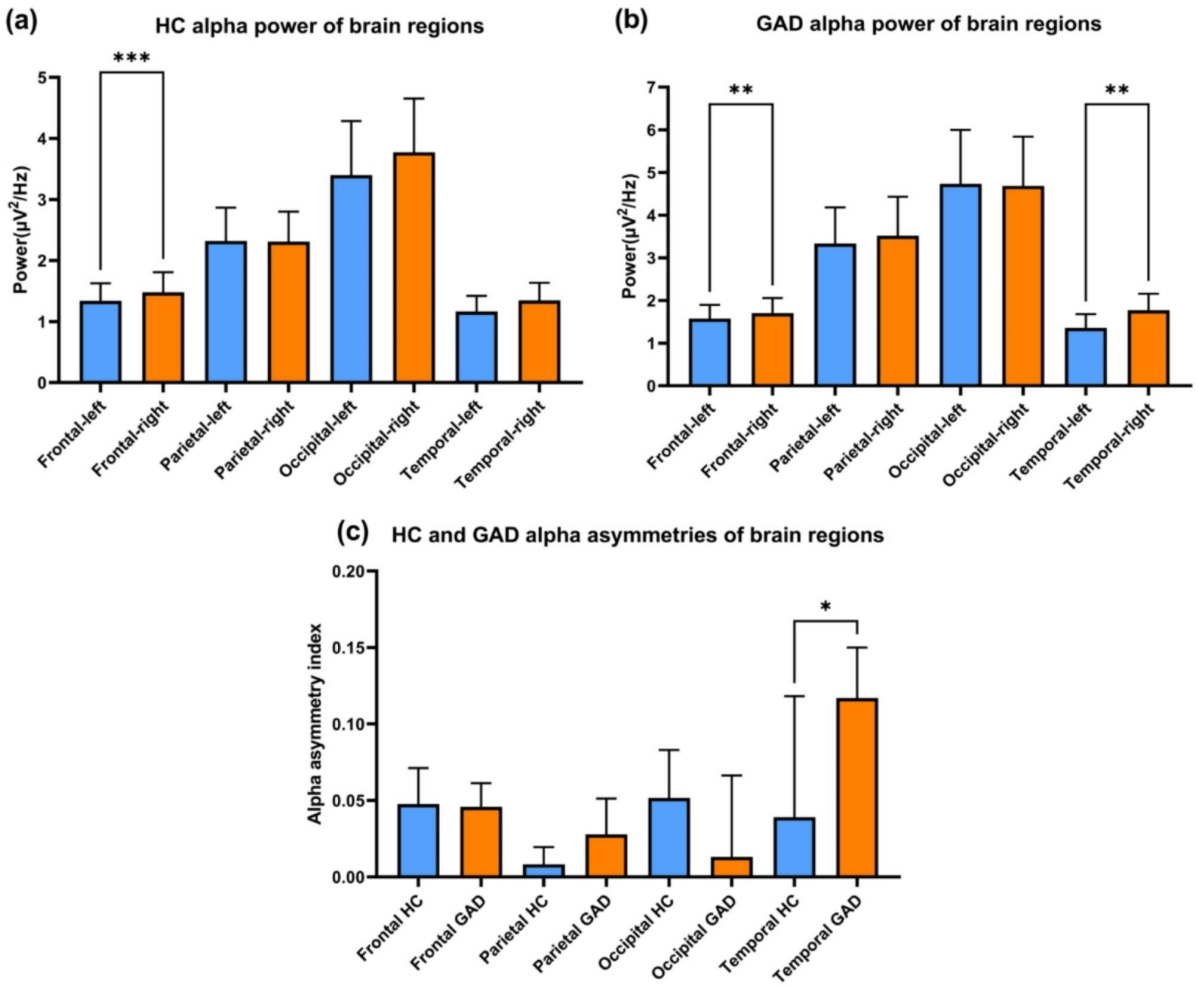
Alpha asymmetry analysis results for the GAD and healthy control groups. (**a**) The average alpha power of the brain regions of HC participants and (**b**) GAD patients. The X-axis represents the brain regions, including the frontal, parietal, occipital, and temporal lobes of the left and right hemispheres. The Y-axis represents the average power density in the alpha band. The error bars represent the 95% confidence intervals. (**c**) The alpha asymmetry indices of brain regions in the HC and GAD groups. The X-axis represents the brain regions and groups. The Y-axis represents the alpha asymmetry index, which was calculated with the laterality coefficient (LC): (A − B)/(A + B), where A and B are the average alpha power densities of channels on the right and left hemispheres, respectively. The error bars represent the 95% confidence intervals.

Additionally, the alpha asymmetry analysis with repeat-measures one-way ANOVA revealed significantly different alpha-band power through channels in both the GAD (F = 34.00, *p* < 0.001, R^2^ = 0.260) and HC (F = 32.17, *p* < 0.001, R^2^ = 0.261) groups. Post-hoc analyses with multiple comparisons revealed significant rightward frontal alpha asymmetry in both the HC (q < 0.001, *p* < 0.001; Fig. 4a) and GAD (q = 0.006, *p* = 0.006; Fig. 4b) groups, as well as significant rightward temporal alpha asymmetry in GAD patients (q = 0.002, *p* = 0.001). When the alpha asymmetry indices of the two groups were compared, the GAD patients presented significantly greater rightward temporal alpha asymmetry (*p* = 0.004, Fig. 4c).

### Functional connectivity analysis

**Fig. 5:**
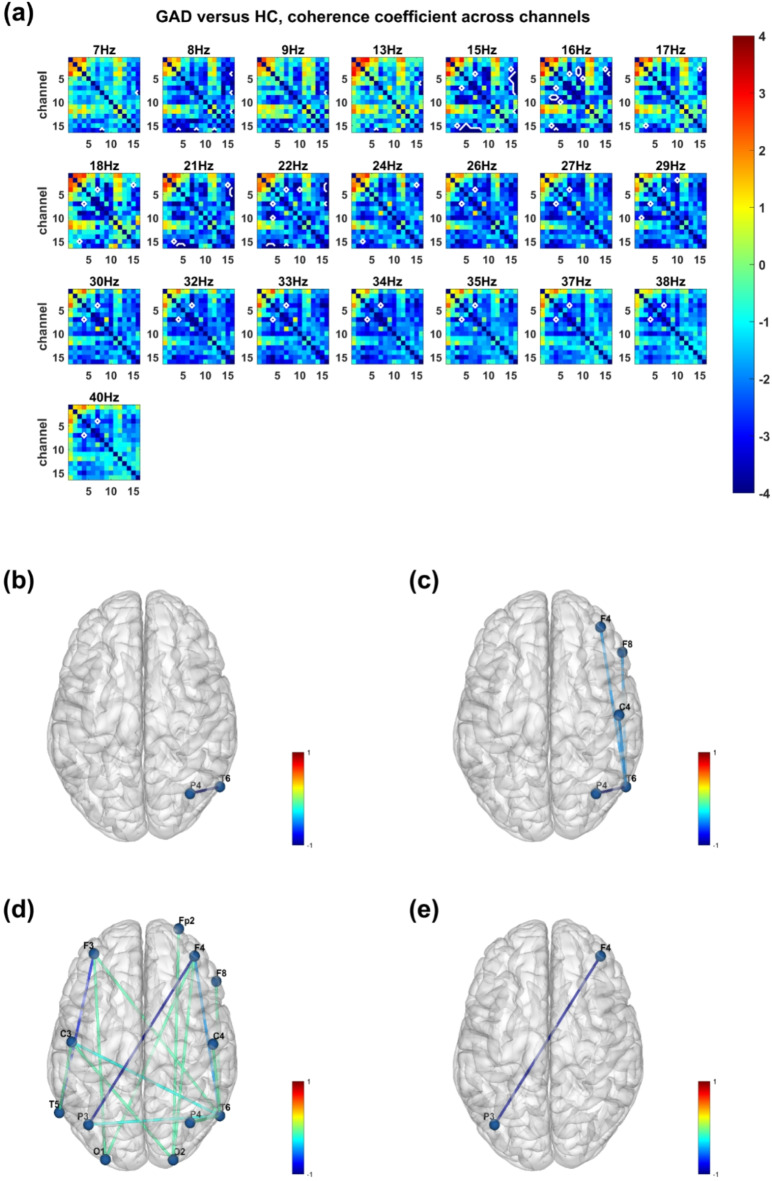
Functional connectivity analysis results between the GAD group and healthy control group via the coherence coefficient algorithm. (**a**) T-statistics of comparing coherence coefficients across channels between the GAD group and the healthy control group. The X- and Y-axes represent the channels. The color bar denotes the t value, with red indicating higher coherence coefficients and blue indicating lower coherence coefficients in the GAD group than in the healthy control group. The white lines highlight the pairs of channels with significant differences between groups after FDR correction (*p* < 0.05). (**b**) The numbers of functional connectivities with significant differences between groups in the theta (4–7 Hz), (**c**) alpha (8–13 Hz), (**d**) beta (14–30 Hz), and (**e**) gamma (31–60 Hz) bands, which were normalized between 0 and 1. The color bar denotes the normalized counts of significant connectivities, with red (positive value) indicating a higher coherence coefficient and blue (negative value) indicating a lower coherence coefficient in the GAD group than in the healthy control group.

The functional connectivity analysis employed two kinds of algorithms: the coherence coefficient and pairwise-phase consistency (PPC). The results of the coherence coefficient algorithm revealed significantly decreased functional connectivity in GAD patients compared with HCs (*p* < 0.05), especially in the alpha and beta bands (Fig. 5a). Specifically, decreased connectivities were observed between the right hemisphere parietal and temporal lobes in the theta (4–7 Hz, Fig. 5b), alpha (8–13 Hz, Fig. 5c), and beta (14–30 Hz, Fig. 5d) bands; between the frontal and temporal lobes in the alpha and beta bands; and between the frontal and occipital lobes in the beta band; as well as between the frontal and parietal lobes in the beta and gamma (31–60 Hz, Fig. 5e) bands.

**Fig. 6:**
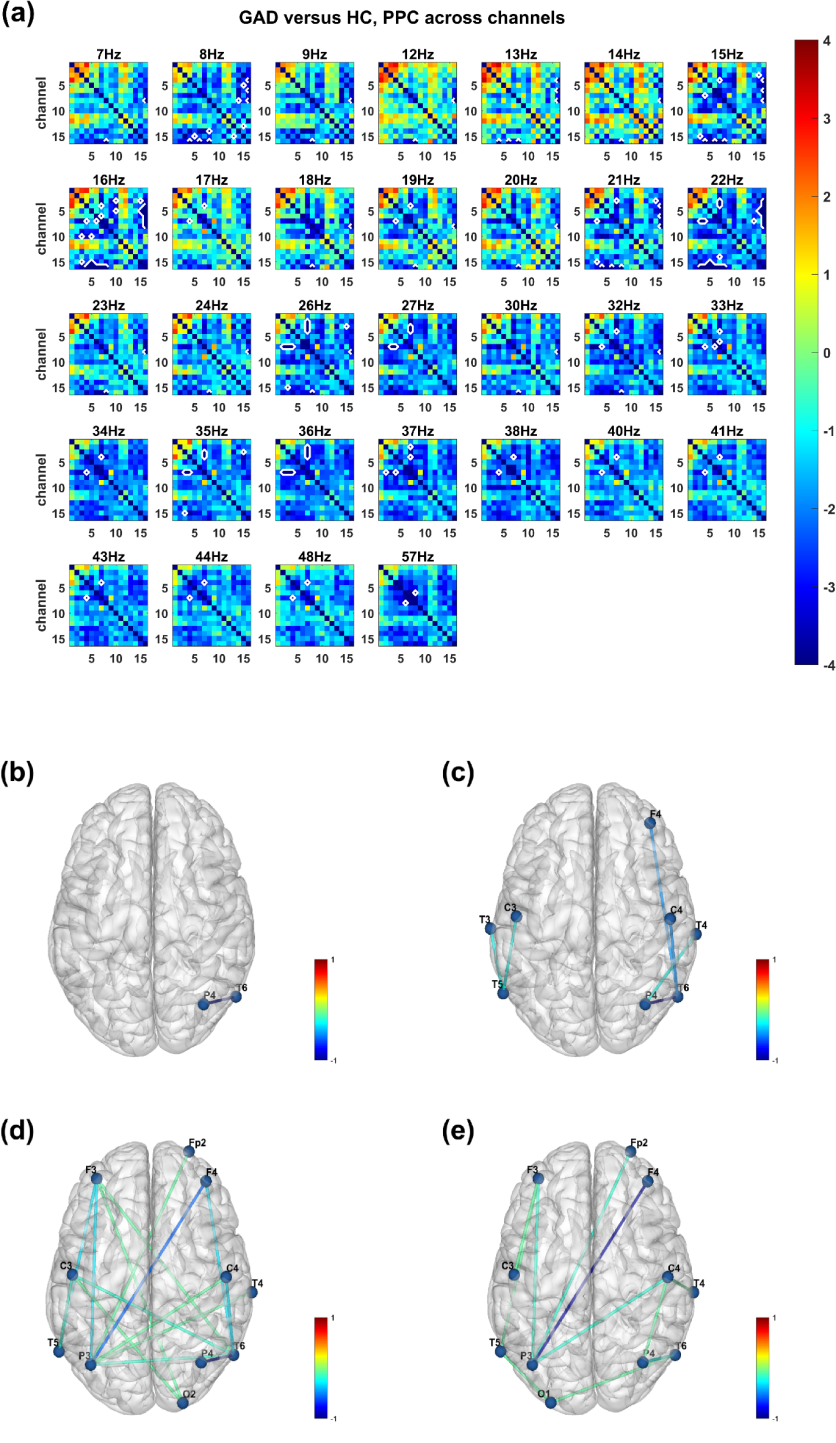
Functional connectivity analysis results between the GAD group and the healthy control group via the pairwise phase consistency (PPC) algorithm. (**a**) T-statistics of comparing PPC across channels between the GAD group and the healthy control group. The X- and Y-axes represent the channels. The color bar denotes the t value, with red indicating a higher PPC value and blue indicating a lower PPC value between channels in the GAD group than in the healthy control group. The white lines highlight the pairs of channels with significant differences between groups after Bonferroni correction (*p* < 0.05). (**b**) The numbers of functional connectivities with significant differences between groups in the theta (4–7 Hz), (**c**) alpha (8–13 Hz), (**d**) beta (14–30 Hz) and, (**e**) gamma (31–60 Hz) bands, which were normalized between 0 and 1. The color bar denotes the normalized numbers of significant connectivities, with red (positive value) indicating a higher PPC and blue (negative value) indicating a lower PPC in the GAD group than in the healthy control group.

The results of the pairwise phase consistency (PPC) algorithm also revealed significant differences between the two groups (*p* < 0.05, Fig. 6a). The results are similar to those of the coherence coefficient method, including decreased connectivities between the right-hemisphere parietal and temporal lobes in the theta (4–7 Hz, Fig. 6b), alpha (8–13 Hz, Fig. 6c), beta (14–30 Hz, Fig. 6d) and gamma (31–60 Hz, Fig. 6e) bands; between the frontal and temporal lobes in the alpha and beta bands; and between the frontal and occipital lobes in the beta band; as well as between the frontal and parietal lobes in the beta and gamma bands.

## Discussion

In this resting-state EEG study, a comparative analysis of the brain activity power spectra and functional connectivities between GAD patients and healthy controls was conducted. The results demonstrate that the brain activity power of GAD patients is higher than that of healthy controls in the beta band. Alpha asymmetry analysis revealed a significantly greater rightward temporal alpha asymmetry in GAD patients. Moreover, there was significantly decreased functional connectivity between several brain regions of GAD patients These findings support our hypothesis that there are abnormal EEG features exist in GAD patients and are discussed in detail below.

The beta rhythm is typically associated with motor control^41,42^, brain alertness levels^43,44^, and the mediation of higher cognitive functions in cognitive processes (e.g., endogenous, top-down component of brain processing)^45,46^. In pathological research, the exacerbation of beta-band activity is usually associated with abnormal behavioral and cognitive changes such as Parkinson’s disease^47^ or social phobia^13^. Our finding of increased beta-band activity in GAD patients aligns with existing research highlighting the important role of the beta rhythm in anxiety^11–14^. These studies indicated increased beta-band activity in patients with anxiety disorders or healthy participants experiencing an induced anxiety state. In conjunction with the results of the present study, the significantly increased beta-band activity in GAD patients suggests abnormal hyperarousal of brain alertness.

EEG resting-state alpha asymmetry is one of the most widely investigated forms of brain lateralization^48^. Previous studies have focused mostly on frontal alpha asymmetry. Greater rightward frontal alpha asymmetry has been revealed in participants with anxious profiles^17^ and in GAD patients^20^. However, although there was significant rightward frontal alpha asymmetry in GAD patients in the present study, it was not significant between the GAD and healthy control groups. In fact, the comparison between groups revealed a greater rightward temporal alpha asymmetry in GAD patients. Temporal lobe alpha asymmetry has been less discussed in previous studies, and some researchers have suggested that it is associated with anxious arousal as a part of parietal-temporal activity^49,50^. As alpha power is inversely related to cortical activity^21^, the greater rightward temporal alpha asymmetry in the present study reflects higher leftward temporal cortical activity in GAD patients, which may be one of the physiological characteristics of GAD patients.

In the present study, temporal lobe EEG features of GAD patients, including decreased ipsilateral frontal–temporal and parietal-temporal functional connectivities in the theta, alpha, and beta bands, were also revealed by the functional connectivity analysis. The temporal lobe plays an important role in emotional regulation and is associated with anxiety symptoms. Previous studies have reported that patients with temporal lobe epilepsy are at high risk of anxiety disorders^51,52^, indicating that temporal lobe dysfunction may be a potential cause of anxiety disorders. Similar to the findings of this study, the decreased functional connectivity between the temporal lobe and other brain regions has also been reported in patients with panic disorder^53^, and in Parkinson’s disease patients with anxiety^54^. The importance of abnormal temporal lobe functions in anxiety disorders could also be supported by the decreased mean temporal lobe volume in patients with panic disorder^55^ and the significant increase in blood flow in the bilateral temporal poles in healthy volunteers during anticipatory anxiety^56^.

The analysis also revealed other abnormal functional connectivities in GAD patients, including decreased frontal‒occipital functional connectivities in the beta band and decreased frontal‒parietal functional connectivities in the beta and gamma bands. The functional connectivities were estimated via two methods, the coherence coefficient and pairwise phase consistency, both of which yielded similar results. These findings suggest that GAD disrupts the functional connectivity between several brain regions in patients. In particular, the findings of decreased functional connectivity between the frontal, parietal, and temporal lobes in resting-state EEG indicate possible dysfunction of a system that plays a major role in regulating resting-state brain activity: the default mode network (DMN). The DMN is a network of brain regions, including the medial prefrontal lobe, posterior cingulate cortex, medial temporal lobe, and other brain areas, that are typically suppressed when an individual is focused on external stimuli and switches to internally focused thought processes in the absence of attention to external stimuli. It is thought to be involved in several higher-order integrative mental functions, including self-reference, social cognition, episodic memory, and emotion regulation. The impairment of the resting-state DMN may be characteristic of a variety of mental disorders, including Alzheimer’s disease, depression, and schizophrenia^57^. In particular, the relationship between impaired DMN and anxiety has also been suggested by previous studies. In participants with trait anxiety, a negative relationship between the resting-state DMN functional connectivity strength and the degree of trait anxiety was observed in both fMRI^58^ and EEG studies^59^. Resting-state hypoconnectivity within DMN regions has also been reported in patients with social anxiety disorder via fMRI^60^. In particular, in previous fMRI studies of GAD patients, patients presented decreased resting-state functional connectivity between the left amygdala and a bilateral region of the rACC^61^; disruptions in the limbic-prefrontal and limbic-default-mode network circuits^62^; and increased DMN functional connectivity in the anterior cingulate cortex (ACC) and bilateral insula, which is correlated with improvements in anxiety following mindfulness-based cognitive therapy^62^. On the basis of the findings of the above studies, a hypothesis is that the decreased EEG functional connectivity between the frontal, parietal, and temporal lobes observed in the present study may be features of DMN dysfunction in resting-state GAD patients. This dysfunction of DMN may also have caused the increased activity we observed in the power spectrum analysis due to the impairment of its inhibitory function. However, considering the insufficient electrode density in this study (e.g., the lack of electrodes on Fz and Pz), the above hypotheses still need further verification.

In conclusion, this study reveals various EEG characteristics of GAD patients compared with those of healthy controls, including increased beta-band activity and greater rightward temporal alpha asymmetry, as well as decreased ipsilateral fronto‒temporal and parieto‒temporal functional connectivities in the lower frequency bands (theta to beta), and decreased frontal‒parietal and frontal‒occipital connectivities in the higher frequency bands (beta to gamma). These results demonstrate excessive resting-state electrophysiological activity and DMN dysfunction in GAD patients, indicating impaired neurological functions, including attention, alertness, and emotion control. Overall, the electrophysiological findings reported in this study have the potential to elucidate the neural mechanisms underlying GAD and provide theoretical support for the identification of electrophysiological biomarkers for GAD diagnosis.

## Limitations

While this study yielded some noteworthy findings, some limitations need to be considered. First, owing to equipment limitations, an EEG system with only 16 electrodes was used in this study. The limited electrode density may have reduced the breadth of the results and limited further analysis. Future research should consider using an EEG system with higher electrode density. Second, this study was solely based on resting-state EEG. Given the significance of the observed results in cognitive processes and the importance of cognitive impairment in anxiety symptoms, further research is planned to focus on cognitive function-related performance and apply task-related EEG (e.g., event-related potentials) in GAD patients. Third, although the HAMD score of GAD patients was controlled below the clinical threshold at enrollment (HAMD < 17), the HAMD score of GAD patients was still significantly higher than that of healthy controls (Table 1). Considering some similarities between the clinical indices of anxiety and depression, the potential confounding effect is difficult to completely exclude in the present study.

Besides the power spectrum, alpha asymmetry, and functional connectivity analyses that have been reported in this article, the present study also conducted several other exploratory analyses, including correlation analysis between the EEG power and HAMA score of GAD patients (Supplementary Fig. S1) and intergroup comparisons of the alpha maximal peak power (Supplementary Fig. S2) and frequency (Supplementary Fig. S3). All of them revealed non-significant results and need to be further verified.

## Electronic supplementary material

Below is the link to the electronic supplementary material.

Supplementary Material 1

## Data Availability

The data that support the findings of this study are not openly available due to reasons of sensitivity and are available from the corresponding author upon reasonable request. The data are located in controlled access data storage at Huzhou Third Municipal Hospital.
