# Supplementary material for: The power spectrum and functional connectivity characteristics of resting-state EEG in patients with generalized anxiety disorder
Source: Sci Rep. 2025 Feb 18;15:5991. doi: 10.1038/s41598-025-90362-z (PMC11836123; doi:10.1038/s41598-025-90362-z)
Supplement: Supplementary file 1 — Supplementary Material 1 [file 41598_2025_90362_MOESM1_ESM.docx]

**T****he Power Spectrum and Functional Connectivity Characteristics of Resting-state EEG in Patients with Generalized Anxiety Disorder**

**SUPPLEMENTARY MATERIALS**

**Hangwei Wang^1,2^, Shaoqi Mou****^3^, Xuedan Pei^4^, Xiaomei Zhang^2^, Shanhong Shen^2^, Jianfeng Zhang^2^, Xinhua Shen^2^, Zhongxia Shen^2,*^**

^1^Key Laboratory of Psychiatry, Huzhou Third Municipal Hospital, the Affiliated Hospital of Huzhou University, Huzhou 313000, People's Republic of China;

^2^Sleep Medical Center, Huzhou Third Municipal Hospital, the Affiliated Hospital of Huzhou University, Huzhou 313000, People's Republic of China;

^3^Qingdao Mental Health Center, Qingdao 266034, People's Republic of China;

^4^Jifu Hospital, Xuzhou 221112, People's Republic of China.

^*^ Corresponding author: snowszx@sina.com

**Correlation analysis between GAD power spectrum and HAMA score**

Based on the power spectrum analysis results, the independent sample Pearson correlation coefficient t-statistics were calculated between the power spectra of the GAD patients and their HAMA-14 scores. Notice that the GAD patients were selected with HAMA-14 scores ≥ 14, thus the distribution of their HAMA-14 scores showed positive skewness (skewness = .746, S.E. = .245) in the current database. The natural logarithms of the original HAMA-14 scores were adopted to correct this skewness (skewness after correction = .216, S.E. = .245). The estimated contributions of irrelevant variables, including age, education, and HAMD-17 scores (anxiety/somatization factor scores were excluded), were removed via a general linear model before calculating the correlation coefficient. The results were multiple comparisons corrected via the cluster-based permutation test, with the significance level of correction set at α=.05 (two-tailed), the distance for defining electrodes as neighbors set at 40 mm, and the number of permutations set at 5000. The result showed a relatively higher but not significant correlation between the power of channel O2 and the HAMA-14 score in the beta and gamma band (25-49 Hz).


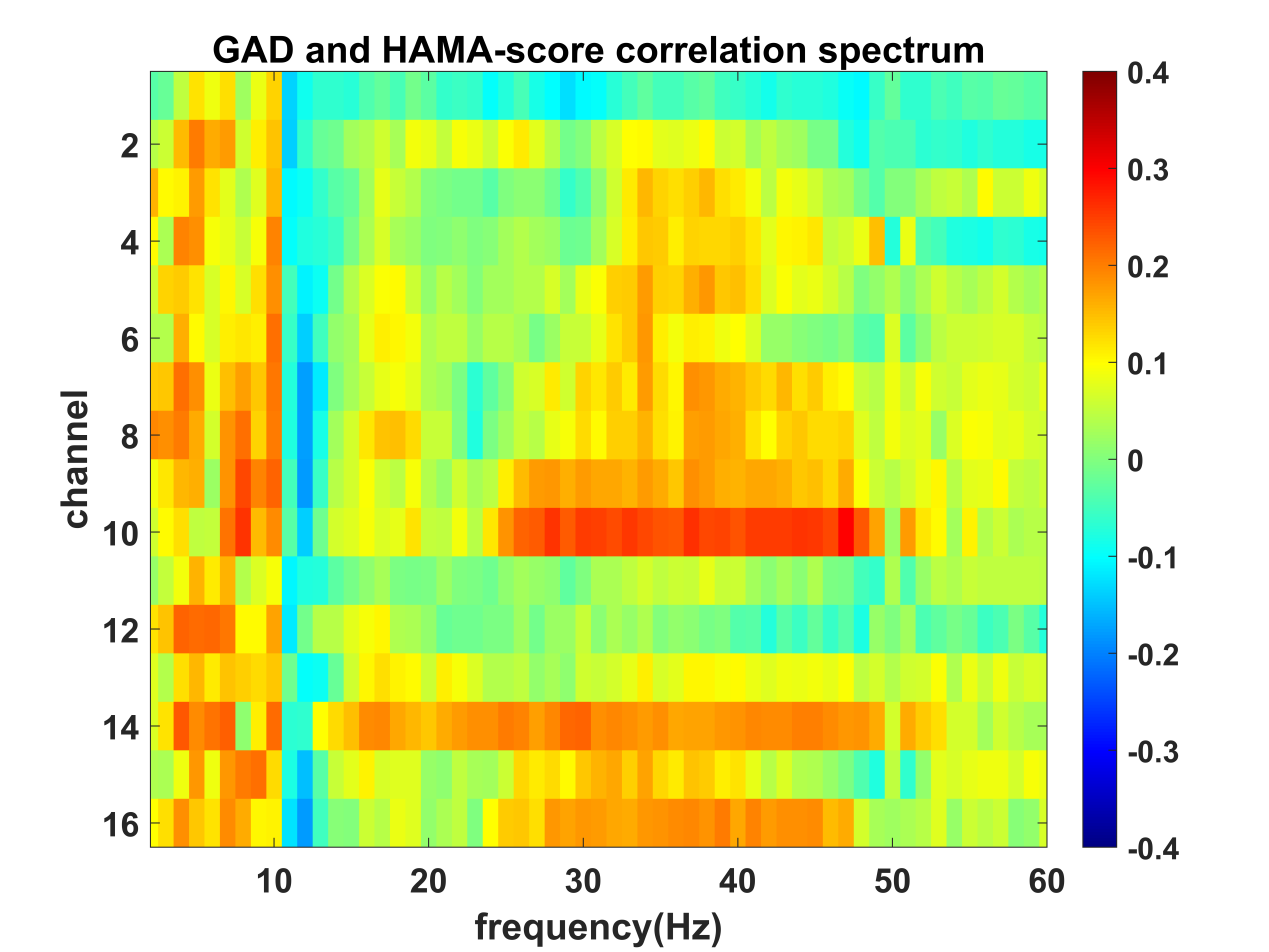


**Supplementary Figure S1.** Correlation coefficient t statistics results between the power spectra of GAD patients and their HAMA-14 scores. The X-axis represents the frequency bins, and the Y-axis represents the channels. The color bar denotes the r-value, with red indicating a positive correlation and blue indicating a negative correlation.

**Alpha maximal peak power and frequency analysis**

As a supplement to power spectrum analysis, t-tests of the alpha maximal peak power and frequency were conducted between the GAD and HC groups to explore potential alpha features. For the definition of alpha maximal peak, power spectra of preprocessed EEG data were calculated via fast Fourier transform (FFT) and averaged across trials within each participant. The frequency resolution of the FFT was set at 0.25 Hz, and the data were tapered via a 4000 ms Hanning window before the FFT. The frequency and power information of the point with the maximal energy in the alpha band (8-13Hz) were recorded for each participant and compared between groups via t-test. The results showed no significant difference between the GAD and HC groups in alpha peak power (fig. S2) or alpha peak frequency (fig. S3).


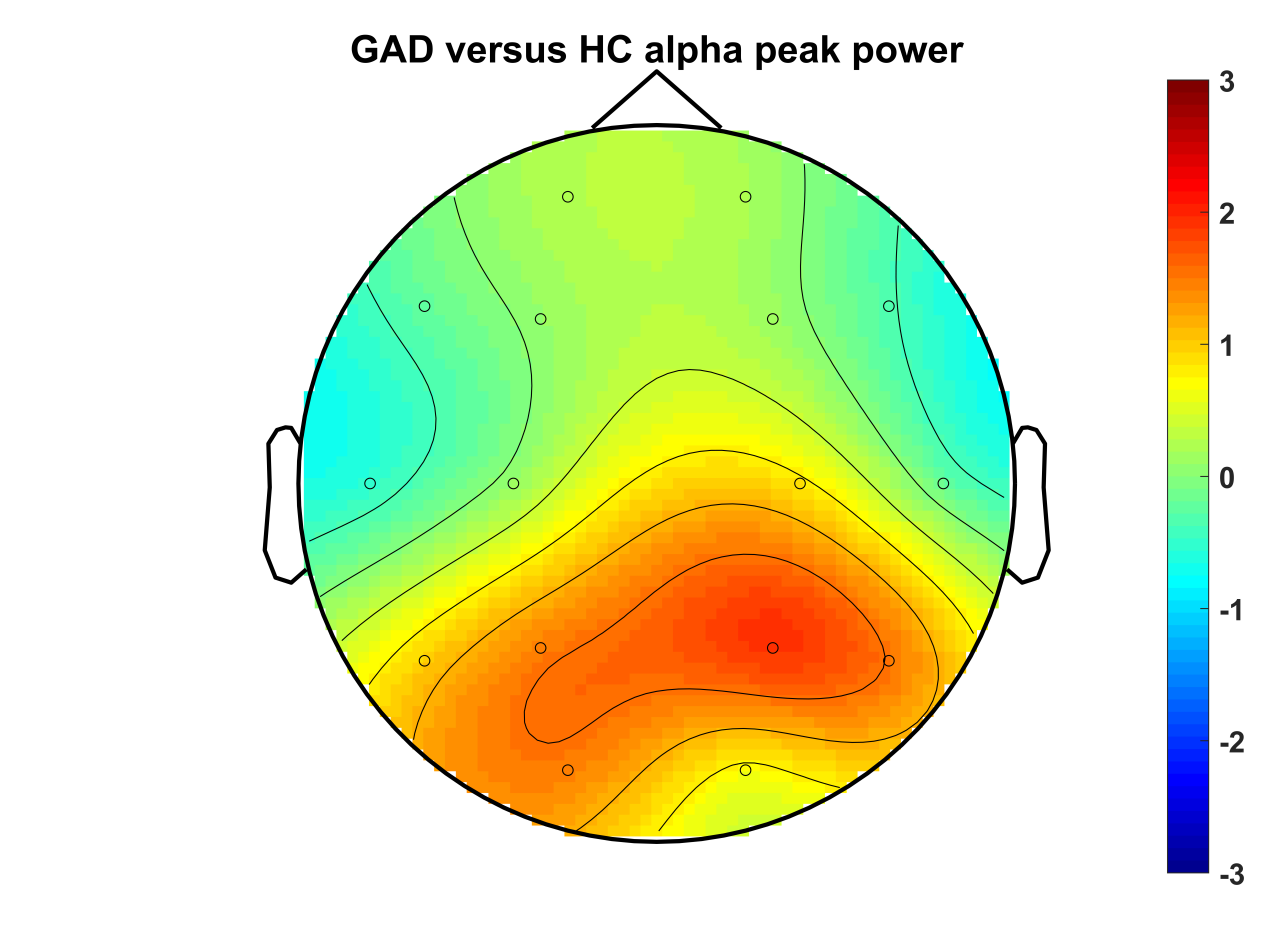


**Supplementary Figure S2**. Topographic distribution of the t-statistics comparing alpha peak power between GAD and HC groups. The color bar denotes the t value, with red indicating higher power and blue indicating lower peak power in the GAD group than in the healthy control group.


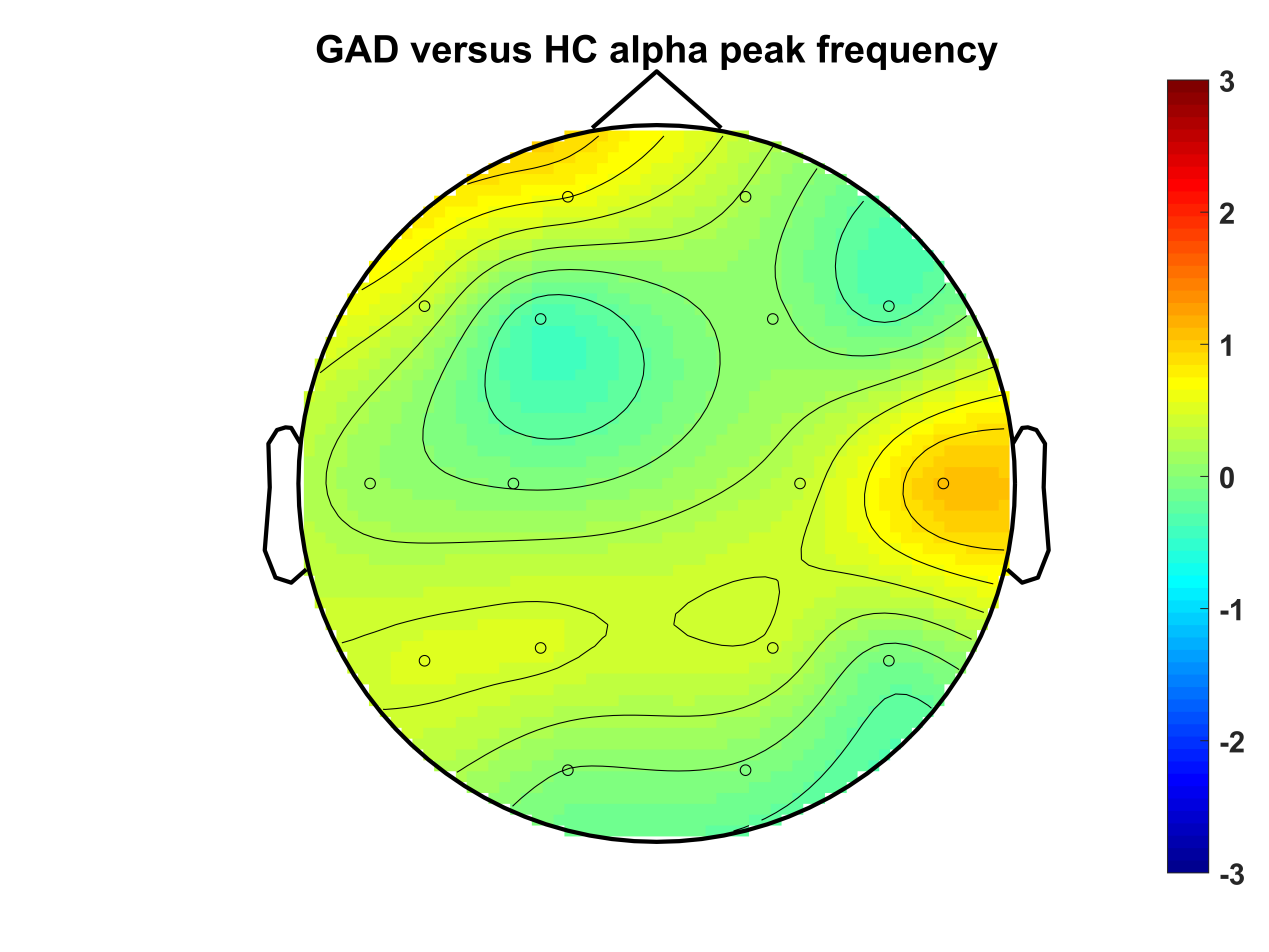


**Supplementary Figure S3.** Topographic distribution of the t-statistics comparing alpha peak frequencies between GAD and HC groups. The color bar denotes the t value, with red indicating higher power and blue indicating lower peak frequencies in the GAD group than in the healthy control group.

**Numerical table of power spectrum analysis result**

To help promote a better understanding of graphical visualized results, this is a numerical form of power spectrum t-test result (Fig 2.a). The rows, which correspond to the x-axis in the graph, represent the frequency bins. The columns, which correspond to the y-axis in the graph, represent the channels. The red numbers, which correspond to the white lines in the graph, denote the point with significant differences between groups after the cluster-based permutation test. The result shows a significantly increased beta (13-27Hz) band activity in the GAD group.

|  | 1Hz | 2Hz | 3Hz | 4Hz | 5Hz | 6Hz | 7Hz | 8Hz | 9Hz | 10Hz | 11Hz | 12Hz | 13Hz | 14Hz | 15Hz |
| --- | --- | --- | --- | --- | --- | --- | --- | --- | --- | --- | --- | --- | --- | --- | --- |
| Fp1 | -1.78 | -0.83 | -0.70 | -0.12 | -0.14 | 0.15 | -0.26 | -0.29 | 0.18 | 1.07 | 1.53 | 1.57 | 1.66 | 1.03 | 0.91 |
| Fp2 | -0.87 | -0.61 | -0.09 | 0.18 | -0.09 | 0.15 | -0.24 | -0.39 | 0.04 | 0.89 | 1.68 | 1.46 | 1.55 | 1.09 | 1.17 |
| F3 | 0.83 | 0.78 | 0.70 | 1.35 | 0.73 | 1.28 | 0.48 | -0.14 | 0.53 | 1.35 | 1.90 | 1.63 | 1.92 | 1.55 | 1.62 |
| F4 | 0.41 | 0.42 | 0.28 | 0.84 | 0.46 | 0.95 | 0.49 | -0.34 | 0.41 | 1.23 | 1.89 | 0.97 | 2.10 | 2.02 | 2.22 |
| C3 | 0.78 | 1.39 | 1.16 | 1.63 | 0.97 | 1.38 | 0.69 | 0.25 | 0.86 | 1.48 | 2.04 | 1.12 | 1.33 | 1.83 | 1.94 |
| C4 | -0.10 | 1.07 | 1.76 | 1.96 | 1.70 | 1.87 | 1.47 | 0.18 | 1.21 | 1.57 | 2.50 | 0.83 | 1.74 | 2.66 | 2.76 |
| P3 | -1.26 | 0.26 | 0.02 | 1.55 | 0.22 | 0.99 | 0.37 | 0.15 | 0.96 | 1.87 | 2.28 | 1.14 | 1.30 | 2.16 | 1.85 |
| P4 | 0.38 | 1.56 | 1.48 | 2.10 | 1.39 | 1.56 | 1.11 | 0.26 | 1.09 | 1.71 | 2.72 | 1.08 | 1.38 | 2.47 | 2.40 |
| O1 | -1.51 | 0.71 | 0.95 | 1.95 | 1.41 | 1.43 | 0.56 | 0.37 | 0.10 | 1.20 | 1.70 | 1.48 | 1.23 | 1.73 | 1.94 |
| O2 | -0.37 | 0.59 | 0.92 | 1.36 | 1.40 | 1.23 | 0.46 | -0.55 | -0.88 | 1.14 | 1.62 | 1.40 | 1.34 | 1.83 | 2.19 |
| F7 | -0.23 | 0.64 | -0.49 | 0.37 | 0.27 | 0.44 | -0.41 | -0.72 | 0.12 | 1.18 | 1.37 | 0.99 | 0.41 | 0.09 | -0.01 |
| F8 | -2.17 | -0.61 | -1.20 | -1.36 | -0.98 | -0.53 | -0.72 | -1.07 | -0.28 | 0.34 | 1.40 | 0.38 | 1.05 | 0.98 | 0.84 |
| T3 | -0.01 | 0.08 | -0.75 | 0.05 | -0.73 | -0.61 | -1.00 | -0.99 | 0.24 | 0.93 | 0.86 | 0.09 | 0.16 | 0.56 | 0.64 |
| T4 | -0.89 | 0.04 | 0.25 | -0.29 | -0.11 | -0.50 | -0.40 | -0.35 | 0.41 | 0.38 | 1.38 | -0.21 | 0.69 | 0.94 | 0.85 |
| T5 | 0.04 | 0.42 | 0.46 | 1.19 | 0.60 | 0.81 | -0.30 | 0.18 | 0.44 | 0.64 | 1.33 | 0.88 | 0.81 | 1.47 | 1.76 |
| T6 | 0.65 | 1.14 | 1.62 | 1.70 | 1.55 | 1.57 | 1.05 | 0.52 | 0.85 | 1.77 | 1.28 | 1.07 | 1.73 | 2.18 | 2.33 |
|  |  |  |  |  |  |  |  |  |  |  |  |  |  |  |  |
|  | 16Hz | 17Hz | 18Hz | 19Hz | 20Hz | 21Hz | 22Hz | 23Hz | 24Hz | 25Hz | 26Hz | 27Hz | 28Hz | 29Hz | 30Hz |
| Fp1 | 1.39 | 1.72 | 1.93 | 2.20 | 1.72 | 1.13 | 1.12 | 0.74 | 0.16 | -0.03 | -0.22 | -0.16 | -0.23 | 0.02 | -0.30 |
| Fp2 | 1.37 | 1.75 | 1.51 | 1.83 | 1.33 | 0.67 | 0.67 | 0.32 | 0.06 | 0.26 | 0.21 | 0.22 | -0.01 | 0.20 | -0.30 |
| F3 | 2.05 | 2.56 | 2.78 | 3.21 | 2.62 | 2.46 | 2.36 | 2.17 | 1.48 | 1.38 | 0.99 | 1.18 | 1.20 | 0.96 | 0.74 |
| F4 | 2.64 | 2.85 | 2.70 | 3.28 | 3.09 | 2.42 | 2.45 | 2.27 | 1.63 | 1.55 | 1.25 | 1.36 | 1.27 | 1.26 | 0.84 |
| C3 | 2.61 | 2.78 | 2.97 | 3.41 | 2.95 | 2.28 | 2.22 | 2.50 | 1.64 | 1.39 | 0.92 | 1.20 | 1.09 | 0.87 | 0.74 |
| C4 | 3.06 | 3.59 | 3.39 | 3.54 | 3.27 | 2.56 | 2.57 | 2.44 | 1.67 | 1.41 | 1.18 | 1.44 | 1.19 | 0.98 | 0.70 |
| P3 | 2.54 | 2.95 | 2.93 | 3.32 | 3.07 | 2.22 | 2.47 | 2.81 | 2.12 | 1.66 | 1.35 | 1.41 | 1.38 | 1.25 | 1.26 |
| P4 | 2.47 | 3.18 | 3.01 | 3.06 | 3.21 | 2.53 | 2.66 | 2.86 | 2.44 | 1.99 | 1.76 | 1.89 | 1.37 | 1.16 | 1.18 |
| O1 | 2.26 | 2.79 | 3.04 | 2.54 | 2.30 | 2.40 | 2.45 | 2.57 | 2.67 | 2.05 | 2.22 | 2.04 | 1.77 | 1.74 | 1.39 |
| O2 | 2.18 | 2.57 | 2.69 | 1.92 | 2.34 | 2.01 | 2.11 | 2.44 | 2.46 | 2.25 | 2.48 | 2.58 | 1.89 | 1.67 | 1.81 |
| F7 | 0.39 | 0.75 | 1.72 | 1.99 | 1.58 | 1.38 | 0.58 | 0.53 | -0.02 | -0.19 | -0.47 | -0.33 | -0.55 | -0.65 | -0.76 |
| F8 | 0.84 | 1.04 | 1.01 | 1.36 | 0.94 | 0.02 | -0.18 | -0.54 | -0.65 | -0.57 | -0.70 | -0.40 | -0.69 | -0.79 | -0.64 |
| T3 | 1.09 | 1.10 | 1.25 | 1.15 | 0.84 | 0.35 | -0.14 | -0.06 | -0.80 | -0.51 | -0.56 | -0.04 | -0.18 | -0.25 | -0.43 |
| T4 | 0.78 | 0.82 | 0.99 | 0.49 | 0.17 | -0.59 | -0.37 | -0.75 | -0.86 | -0.81 | -0.87 | -0.82 | -1.12 | -1.19 | -1.24 |
| T5 | 2.15 | 2.14 | 2.38 | 2.12 | 1.60 | 1.20 | 1.16 | 1.32 | 0.85 | 0.87 | 0.87 | 0.95 | 0.43 | 0.46 | 0.53 |
| T6 | 2.33 | 2.51 | 2.70 | 2.49 | 2.25 | 1.70 | 1.46 | 1.73 | 1.27 | 1.26 | 1.34 | 1.81 | 1.11 | 1.01 | 1.49 |
|  |  |  |  |  |  |  |  |  |  |  |  |  |  |  |  |
|  | 31Hz | 32Hz | 33Hz | 34Hz | 35Hz | 36Hz | 37Hz | 38Hz | 39Hz | 40Hz | 41Hz | 42Hz | 43Hz | 44Hz | 45Hz |
| Fp1 | -0.88 | -0.85 | -1.10 | -1.20 | -1.00 | -1.27 | -1.02 | -1.29 | -1.51 | -1.47 | -1.48 | -1.44 | -1.59 | -1.50 | -1.53 |
| Fp2 | -0.47 | -0.66 | -0.90 | -0.85 | -0.65 | -0.96 | -0.94 | -1.26 | -1.39 | -1.54 | -1.53 | -1.46 | -1.74 | -1.63 | -1.61 |
| F3 | 0.35 | -0.04 | -0.34 | -0.47 | -0.19 | -0.34 | -0.45 | -0.76 | -0.97 | -1.31 | -1.39 | -1.47 | -1.73 | -1.67 | -1.65 |
| F4 | 0.42 | 0.40 | 0.40 | 0.68 | 1.34 | 1.09 | 0.67 | 0.14 | -0.28 | -0.55 | -0.82 | -1.15 | -1.37 | -1.47 | -1.43 |
| C3 | 0.46 | -0.02 | 0.05 | -0.12 | 0.10 | -0.11 | -0.25 | -0.74 | -0.91 | -1.11 | -1.14 | -1.37 | -1.63 | -1.54 | -1.61 |
| C4 | 0.51 | 0.09 | -0.14 | 0.05 | 0.66 | 0.14 | 0.05 | -0.47 | -0.69 | -0.91 | -0.93 | -1.15 | -1.34 | -1.33 | -1.37 |
| P3 | 1.11 | 0.56 | 0.82 | 0.33 | 0.97 | 0.12 | 0.04 | -0.44 | -0.87 | -1.08 | -1.15 | -1.36 | -1.69 | -1.60 | -1.68 |
| P4 | 1.19 | 0.69 | 0.73 | 0.42 | 1.16 | 0.20 | 0.00 | -0.44 | -0.74 | -1.04 | -1.15 | -1.27 | -1.58 | -1.50 | -1.56 |
| O1 | 1.19 | 1.24 | 1.31 | 1.10 | 1.41 | 1.25 | 0.78 | 0.91 | 0.34 | 0.10 | -0.51 | -1.02 | -1.29 | -1.36 | -1.67 |
| O2 | 1.80 | 1.50 | 1.79 | 1.41 | 1.79 | 1.35 | 1.12 | 1.26 | 0.86 | 0.28 | -0.43 | -0.84 | -1.24 | -1.55 | -1.75 |
| F7 | -1.13 | -1.21 | -1.18 | -0.98 | -0.78 | -0.89 | -1.00 | -1.14 | -1.34 | -1.57 | -1.55 | -1.44 | -1.64 | -1.57 | -1.62 |
| F8 | -1.29 | -1.44 | -1.79 | -1.65 | -1.35 | -1.47 | -1.68 | -1.81 | -1.82 | -1.95 | -1.76 | -1.71 | -1.83 | -1.72 | -1.70 |
| T3 | -0.07 | -0.09 | -0.25 | -0.52 | -0.29 | -0.16 | -0.43 | -0.50 | -0.80 | -0.98 | -1.05 | -1.23 | -1.53 | -1.45 | -1.48 |
| T4 | -0.97 | -0.86 | -1.07 | -1.06 | -0.80 | -0.97 | -0.97 | -1.21 | -1.34 | -1.26 | -1.32 | -1.27 | -1.35 | -1.36 | -1.30 |
| T5 | 0.53 | 0.34 | 0.58 | 0.19 | 0.57 | 0.09 | -0.09 | -0.34 | -0.54 | -0.90 | -1.03 | -1.32 | -1.60 | -1.47 | -1.62 |
| T6 | 1.62 | 1.31 | 1.02 | 0.83 | 1.07 | 0.69 | 0.60 | 0.35 | -0.13 | -0.51 | -0.82 | -1.07 | -1.36 | -1.50 | -1.60 |
|  |  |  |  |  |  |  |  |  |  |  |  |  |  |  |  |
|  | 46Hz | 47Hz | 48Hz | 49Hz | 50Hz | 51Hz | 52Hz | 53Hz | 54Hz | 55Hz | 56Hz | 57Hz | 58Hz | 59Hz | 60Hz |
| Fp1 | -1.67 | -1.61 | -1.61 | -1.51 | -2.65 | -1.76 | -1.75 | -1.62 | -1.59 | -1.75 | -1.70 | -2.02 | -2.01 | -2.40 | -2.59 |
| Fp2 | -1.75 | -1.61 | -1.65 | -1.45 | -2.06 | -1.74 | -1.83 | -1.73 | -1.67 | -1.77 | -1.70 | -1.95 | -1.80 | -1.84 | -1.68 |
| F3 | -1.81 | -1.71 | -1.76 | -1.52 | -1.59 | -1.81 | -1.91 | -1.81 | -1.78 | -1.85 | -1.83 | -1.92 | -1.61 | -1.50 | -1.14 |
| F4 | -1.58 | -1.66 | -1.69 | -1.80 | -1.69 | -1.83 | -1.94 | -1.73 | -1.73 | -1.90 | -1.70 | -1.89 | -1.88 | -1.57 | -1.21 |
| C3 | -1.74 | -1.68 | -1.74 | -1.55 | -1.89 | -1.77 | -1.90 | -1.82 | -1.76 | -1.86 | -1.81 | -1.95 | -1.62 | -1.26 | -1.08 |
| C4 | -1.44 | -1.51 | -1.51 | -1.23 | -2.10 | -1.50 | -1.76 | -1.67 | -1.60 | -1.78 | -1.63 | -1.95 | -1.82 | -1.75 | -1.50 |
| P3 | -1.74 | -1.74 | -1.77 | -1.62 | -2.50 | -1.88 | -1.98 | -1.91 | -1.79 | -1.94 | -1.88 | -2.17 | -2.09 | -2.18 | -2.04 |
| P4 | -1.60 | -1.64 | -1.66 | -1.56 | -2.34 | -1.77 | -1.84 | -1.77 | -1.66 | -1.87 | -1.71 | -2.08 | -2.02 | -2.11 | -2.00 |
| O1 | -1.73 | -1.76 | -1.73 | -1.65 | -2.45 | -1.89 | -2.00 | -2.00 | -1.88 | -1.92 | -1.89 | -2.12 | -2.08 | -2.08 | -1.90 |
| O2 | -1.87 | -1.85 | -1.90 | -1.76 | -2.69 | -2.02 | -2.15 | -2.18 | -2.06 | -2.15 | -2.01 | -2.41 | -2.52 | -2.62 | -2.55 |
| F7 | -1.77 | -1.62 | -1.69 | -1.41 | -2.01 | -1.68 | -1.79 | -1.64 | -1.75 | -1.75 | -1.62 | -1.84 | -1.62 | -1.46 | -0.96 |
| F8 | -1.77 | -1.72 | -1.67 | -1.56 | -2.63 | -1.79 | -1.88 | -1.83 | -1.73 | -1.89 | -1.87 | -2.09 | -2.11 | -2.26 | -2.34 |
| T3 | -1.67 | -1.58 | -1.63 | -1.41 | -2.36 | -1.64 | -1.78 | -1.63 | -1.71 | -1.72 | -1.66 | -2.00 | -1.87 | -1.97 | -2.15 |
| T4 | -1.34 | -1.38 | -1.39 | -1.29 | -1.63 | -1.47 | -1.57 | -1.46 | -1.35 | -1.52 | -1.39 | -1.58 | -1.52 | -1.62 | -1.71 |
| T5 | -1.61 | -1.72 | -1.71 | -1.51 | -2.45 | -1.77 | -1.89 | -1.79 | -1.84 | -1.86 | -1.88 | -2.12 | -1.99 | -2.08 | -2.22 |
| T6 | -1.74 | -1.70 | -1.62 | -1.53 | -2.47 | -1.79 | -1.84 | -1.91 | -1.77 | -1.96 | -1.83 | -1.97 | -2.12 | -2.18 | -2.45 |

**Supplementary Table S1.** T-statistics of comparing power spectra between GAD and HC groups. The rows represent the frequency bins, the columns represent the channels. Red numbers denote the point with significant difference between groups after the cluster-based permutation test.
